# Changes in mechanical properties at the muscle level could be detected by Nakagami imaging during *in-vivo* fixed-end contractions

**DOI:** 10.1371/journal.pone.0308177

**Published:** 2024-09-13

**Authors:** Andrea Monte, Po-Hsian Tsui, Paola Zamparo

**Affiliations:** 1 Department of Neurosciences, Biomedicine and Movement Sciences, University of Verona, Verona, Italy; 2 Department of Medical Imaging and Radiological Sciences, College of Medicine, Chang Gung University, Taoyuan, Taiwan; New Jersey Institute of Technology, UNITED STATES OF AMERICA

## Abstract

In this study, we investigated the capability of the Nakagami transformation to detect changes in vastus lateralis muscle-tendon stiffness (*k*) during dynamic (and intense) contractions. *k* was evaluated in eleven healthy males using the gold-standard method (a combination of ultrasound and dynamometric measurements) during maximal and sub-maximal voluntary fixed-end contractions of the knee extensors (20, 40, 60, 80, and 100% of maximum voluntary force), while Nakagami parameters were analysed using the Nakagami transformation during the same contractions. Muscle-belly behaviour was investigated by means of B-mode ultrasound analysis, while Nakagami parameters were obtained in post-processing using radiofrequency data. *k* was calculated as the slope of the force-muscle-belly elongation relationship. Three contractions at each intensity were performed to calculate the intra-trial reliability and the coefficient of variation (CV) of the Nakagami parameters. At all contraction intensities, high values of intra-trial reliability (range: 0.92–0.96) and low CV (<9%) were observed. *k* and Nakagami parameters increased as a function of contraction intensity, and significant positive correlations were observed between these variables. These data suggest that changes in mechanical properties (e.g., stiffness) at the muscle level could be investigated by means of Nakagami parameters.

## Introduction

Muscle-tendon stiffness is a mechanical property of the muscle-tendon unit that influences force transmission as well as the storage-restitution of elastic energy, playing an important functional role both in health and disease. Changes in muscle-tendon stiffness have been observed after training and detraining [1], as a function of aging [2], in consequence of musculoskeletal disorders [3] or neurological diseases [4].

Several methods were proposed in the literature to characterize the stiffness of isolated tissues in vivo. The most commonly used method for the *in-vivo* evaluation of muscle-tendon stiffness requires a combination of dynamometric and ultrasound measurements (e.g. [5–7]), which is impractical for routine or clinical use. Hence, new techniques able to detect and quantify changes in muscle-tendon stiffness are of interest to both scientists and clinicians.

Compared to conventional B-mode ultrasound, quantitative ultrasound (QUS) techniques offer the advantage to directly access the radio-frequency (RF) data, allowing to extract objective and quantitative metrics for tissue characterization. Indeed, QUS techniques were exploited, with a certain degree of success, to improve medical diagnostics of different soft tissues [8]. For example, tissue characterization based on QUS techniques is utilized in the diagnosis of breast and prostate cancer [9]. Other studies utilized strain elastography imaging from the RF signal to investigate the different stages of liver fibrosis [10, 11].

Basically, QUS techniques use the echo amplitude distribution of the raw signal to characterize the tissues: different scattering structures result in different backscattered statistics [8]. In musculoskeletal tissues, the most common QUS technique is called shear-wave-elastography (SWE). SWE uses a radiation pulse sequence to generate shear waves, causing transient displacements that propagate along the tissues. Since shear waves propagate faster through stiffer tissue, SWE was used to investigate the tissue’s intrinsic elastic characteristics (e.g., muscle, tendons, soft tissues) in different populations [12–14]. However, SWE is not usually implemented in commercial ultrasound apparatuses since it requires a specific probe and specific system characteristics.

Another way of describing the mechanical characteristics of soft tissues is the Nakagami distribution which is a statistical model able to identify (based on grayscale information) the scattering characteristics within a tissue [13, 15, 16] utilizing shape parameters that depend on the shape of the envelope distribution. Specifically, the Nakagami parameters can be estimated by use of the statistical moments of backscattered envelope signals, and then utilized to measure the backscattered statistics pattern for evaluating tissue homogeneity [13, 15, 16]. Therefore, Nakagami transformation could give insights into mechanical tissue properties, but provide different information compared with SWE.

The Nakagami parameters have an outstanding ability to detect the variation of the scatter concentration and provide information on scatter arrangements and concentrations to complement B-scan for tissue characterization in clinical applications [17]. For example, Nakagami imaging was used, alone or in combination with shape parameters and texture, to classify benign and malignant breast tumors [18–20] and to assess liver fibrosis in rats [11]. The shape parameter (*m*), related to the shape, size and concentration of tissue scatterers could indeed provide useful information on the tissue microstructure [18] and could give insights into mechanical tissue proprieties [21].

The Nakagami parameters were also used for monitoring the progression of Duchenne dystrophy in ambulatory patients and for predicting ambulation loss [13, 22]. Since the severity of Duchenne dystrophy correlates with muscle stiffness [13], it is possible to hypothesize that changes in Nakagami parameters could (at least partially) reflect changes in tissues’ mechanical properties (i.e., stiffness). If this were the case, QUS techniques based on Nakagami distribution may prove useful for evaluating the mechanical properties of the muscle-tendon unit, providing important information for clinicians and researchers alike.

Hence, the aim of this study was to test and validate the Nakagami transformation as a technique able to detect changes in muscle-tendon unit stiffness. To this aim, we evaluated muscle-tendon stiffness *in-vivo* by means of the common method (a combination of dynamometric and B-mode ultrasound measurements) and by means of the quantitative-ultrasound technique based on Nakagami parameters during a series of maximal and sub-maximal voluntary fixed-end contractions of the knee extensors.

## Materials and methods

### Study population

Eleven healthy males (37±7 years, 76±5 kg, 178±4 cm) were recruited for the experiments. The participants were physically active (2 or 3 training sessions per week of running/cycling) and did not report neuromuscular injuries. The sample size was estimated based on changes in muscle stiffness during contraction of the knee extensor muscles at different force levels [23]. To obtain this sample size we used G*Power, adopting an alpha level of 0.05, an effect size of 0.75, and a statistical power of 0.8.

The Institutional Review Board of the University of Verona approved the experimental protocol (2022-UNVRCLE-0580578), and all participants signed a written informed consent.

### Protocol

Each participant was involved in one single session. After preparation, warm-up (two contractions at about 20, 50 and 80% of the hypothetical maximum contraction intensity), and familiarisation with the equipment, the participants were asked to perform a series of maximal and sub-maximal voluntary fixed-end contractions of the knee extensors. Participants were seated on a custom-made chair that allowed the assessment of the right knee extensors. Straps were fastened across the chest and hips to avoid lateral and frontal displacements. The participant’s knee and hip were flexed at 90°. The external force generated by the knee extensors was recorded by means of a strain gauge. Ultrasound data were acquired in B-mode and RF-mode simultaneously to record the vastus lateralis (VL) fascicle and aponeuroses behaviour (B-mode) as well as the RF-data of the investigated region (RF-mode, see below for further details).

### Data collection

An ankle strap was placed at the level of the lateral malleolus and was connected to a strain gauge (System Pese, Milan, Italy; linear response 1500 N) by means of an inextensible cable, perpendicular to the tibia. The force signal was amplified (x1000) and sampled at 1500 Hz using an external A/D converter (PowerLab, ADInstruments) and recorded with a personal PC using LabChart 7.

After a warm-up, based on a series of sub-maximal isometric contractions, the participants were asked to perform three maximum voluntary fixed-end contractions of the knee extensors, with 2 min of recovery in between, to record maximum voluntary force (F_max_). An operator then calculated 5 different sub-maximal contraction intensities (20, 40, 60 and 80% of the individual F_max_) and the participants were asked to perform three voluntary contractions at each sub-maximal intensity, in a randomised order, with 2 min of recovery in between. During each contraction, the participants were instructed to contract their muscles from 0 intensity to the desired one in approximately 2 seconds, to maintain the target intensity for 1 second and then release; real-time biofeedback was provided on PC. A total of 15 contractions (3 maximal and 12 sub-maximal) were then recorded for each subject.

B-mode and Radiofrequency (RF)-mode ultrasound data were acquired with a portable ultrasound scanner (ArtUs, Telemed) and a 4.5 cm linear transducer (L15-7H40-A5) with a central frequency of 15 MHz, 192 elements, a speed of sound of 1540 m/s, a bandwidth of -6dB and a pulse length of 0.7 mm. The probe was positioned approximately at 50% of the femoral length and aligned on the muscle belly to have a clear image of the perimysial connective intramuscular tissue (e.g., indicative of the muscle fascicle structure). This probe location was adopted since VL middle region best reflects the contribution of the entire muscle in determining the mechanical output. Data acquisition was performed using a dedicated software interface developed by the company. The interface allowed to set the US scanner parameters, such as power, depth, focus, transmission frequency, size and position of the acquisition window and to collect RF and B-mode data for off-line analyses, simultaneously. For each contraction, B-mode and RF-data were acquired during the entire contraction period. Raw data consisted of 192 backscattered radio-frequency signals (for RF-data analysis) and a B-mode video (for B-mode analysis). B-mode image and RF-data consisting of 192 backscattered radio-frequency (RF) signals (i.e., scan lines) were exported in DICOM and MAT files, respectively. The off-line analysis was conducted using custom-developed programs in MATLAB (v2021b).

### Data analysis

#### Knee extensors (internal) force

The external force measured by the strain gauge was converted in external moment by knowing the lower-leg length of each participant (e.g., external moment arm). The internal force generated by the knee extensors was then calculated as the ratio between the external moment and the internal patellar tendon lever arm; the latter was set equal to 4.3 cm, according to the equation proposed by [24] based on a knee angle of 90°, as in our experiments. The internal force generated by each participant at all the investigated contraction intensities was expressed in absolute (N) and relative values (as %F_max_).

#### B-mode ultrasound analysis

A validated automatic tracking algorithm was used to quantify muscle thickness (MT) and pennation angle (PA) frame by frame [25]; this analysis was applied to the maximal isometric contractions only. Muscle thickness was defined as the distance between proximal and distal aponeuroses, while pennation angle as the angle between the collagenous tissue and the deep aponeurosis. At the end of the auto-tracking, every frame of the tracked parameters was visually examined to check the algorithm accuracy. Whenever MT or PA were deemed inaccurate, the two points defining the muscle fascicles were manually repositioned. Fascicle length (FL) and muscle belly length (ML) were then calculated using a classical trigonometric function:

FL=MT/(senPA),andML=FL⋅(cosPA) Eq 1

Since the tendon is at least partially in series with the aponeuroses [26], the ML changes (ΔML) could be affected by the mechanical characteristics of the patellar tendon. For this reason, in the current study, we refer to the stiffness of the muscle-tendon complex.

**Fig 1:**
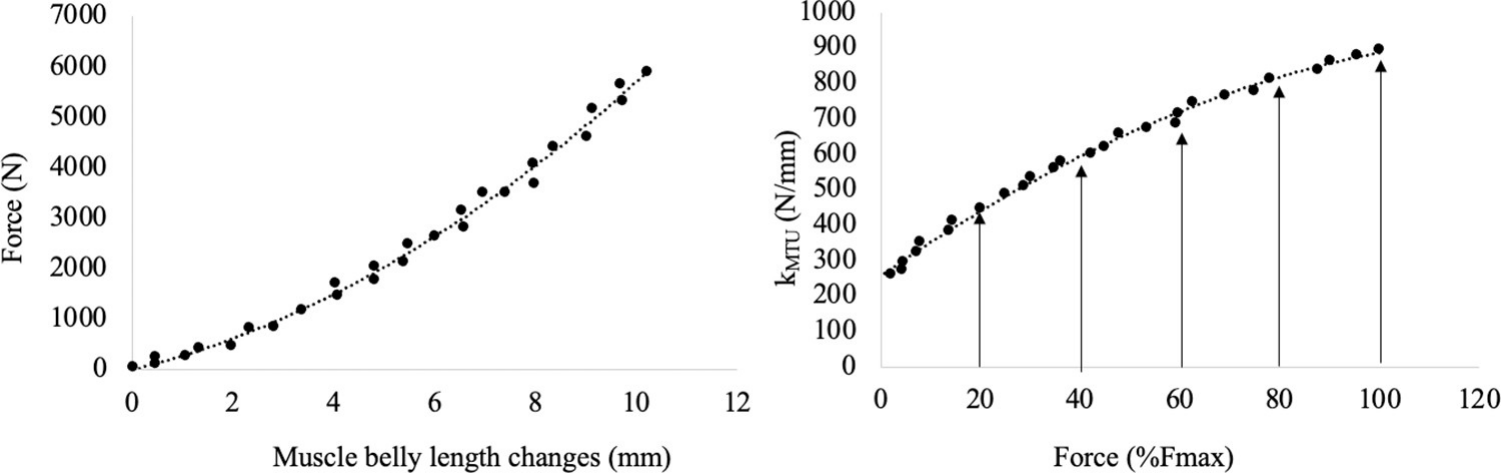
Force-muscle-belly length relationship (left panel) and *k*-force relationships (right panel) for a typical subject. *k* was calculated point-by-point as the ratio between force and muscle-belly length changes. Finally, the *k* values corresponding to selected contraction intensities (arrows at 20, 40, 60, 80 and 100% of the individual F_max_) were recorded and utilised in further analysis.

Muscle-tendon stiffness (*k*) was calculated point-by-point as the ratio between force and muscle-belly length changes: *k* = ΔF/ΔML [27]. Finally, the *k* values corresponding to each contraction intensity (20, 40, 60, 80 and 100% of the individual F_max_) were utilised as the “gold-standard” values for the following analysis (see Fig 1).

#### Quantitative RF- ultrasound analysis

Raw RF-data, collected in the same portion of the muscle belly where the stiffness was evaluated, were elaborated to obtain the Nakagami image. Nakagami distribution, indeed, represents a classical backscattered statistics of the ultrasound echoes intensity [28].

**Fig 2:**
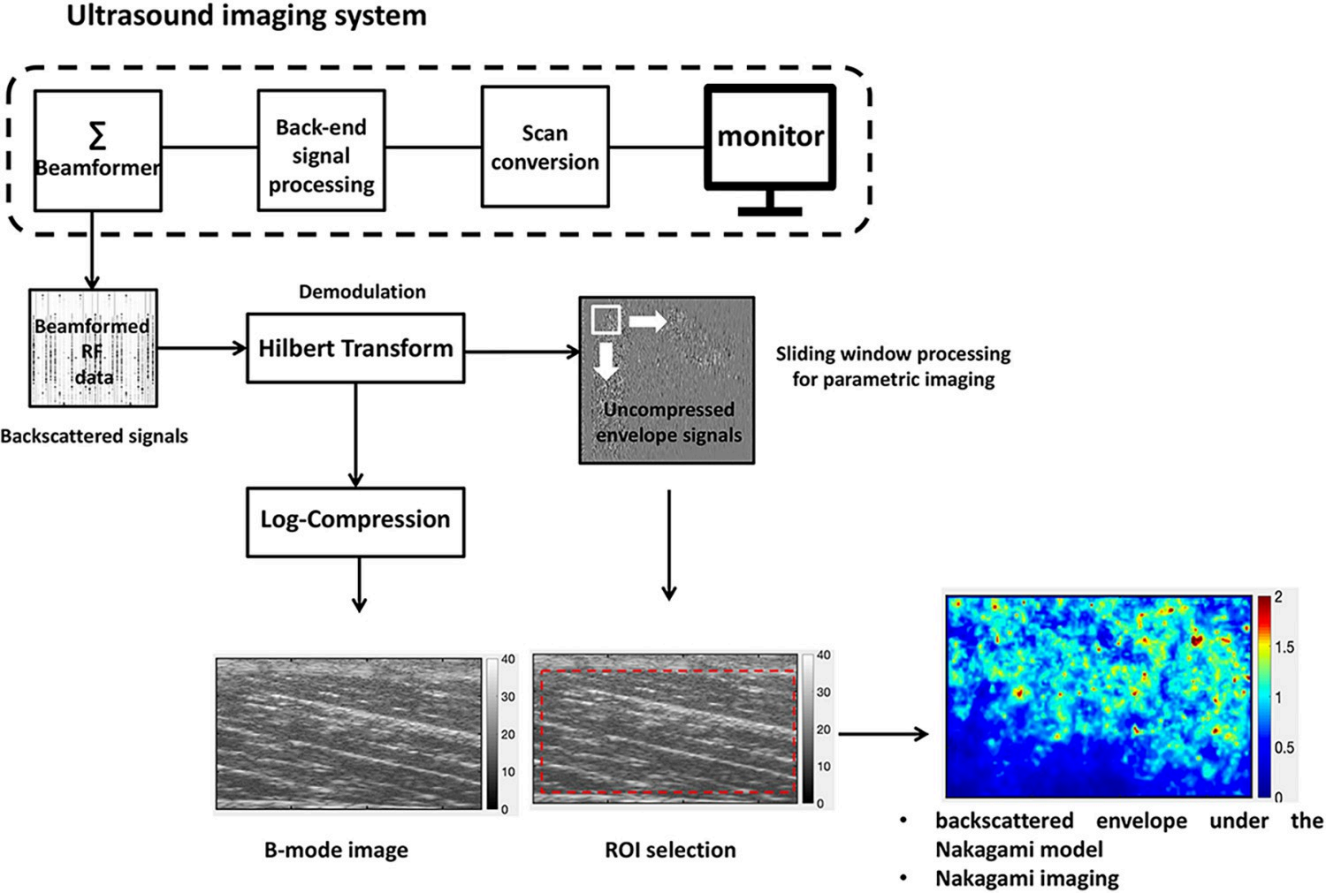
Schematic representation of the basic steps for RF data analysis. Raw data were used to obtain the envelope image by using the absolute value of the Hilbert Transform. Then, log-compressed envelope was used to obtain B-mode images. The uncompressed envelope image was utilized to identify the region of interest (ROI). The Nakagami parameter was calculated as the mean value of each pixel within the ROI.

Hilbert Transform was used to obtain the envelope image of the raw data by means of the absolute value of the backscattered signal. A dynamic range of 40 dB was used to reconstruct the B-mode images by using a log-compressed envelope statistic. The uncompressed envelope image was utilized to identify the region of interest (ROI). The ROI was manually set by an expert operator: it consisted in the entire muscle belly, aponeuroses excluded (see Fig 2).

The Nakagami parameter was calculated as the mean value in pixel within the ROI. More specifically, the distribution of the backscattered envelope *r* under the Nakagami model was calculated as:

$$f(r)=\frac{2m^{m}r^{2m-1}}{\Gamma (m)\Omega^{m}}\exp\left(-\frac{m}{\Omega }r^{2})U(r\right)$$ Eq 2

where Γ and U are the gamma and unit step functions, respectively and E represents the statistical mean. The scaling parameter Ω can be calculated as: E(R^2^), while the Nakagami parameter *m* associated with its distribution can obtained as:

$$m=\frac{{\left[E(R^{2})\right]}^{2}}{E(R^{4})-{\left[E(R^{2})\right]}^{2}}$$ Eq 3

where R is the amplitude of the envelope of the ultrasound signals. As reported by Weng et al. [22]: *“The Nakagami parameter can be interpreted as follows*: *(i) m < 0*.*5 is a Nakagami-gamma distribution (few scatterers with gamma-distributed scattering cross-sections in the resolution cell)*, *(ii) 0*.*5 ≤ m ≤ 1 is a pre-Rayleigh distribution (few scatterers or strong scatterers mixed with randomly distributed scatterers in the resolution cell)*, *(iii) m = 1 is a Rayleigh distribution (a large number of randomly distributed scatterers in the resolution cell)*, *and (iv) m > 1 is a post-Rayleigh distribution (periodically located scatterers and randomly distributed scatterers in the resolution cell)*. *Therefore*, *the Nakagami parameter enables the quantification of echo amplitude distributions with specific physical meanings associated with scattering structures”* [22].

To obtain high-quality images and visualization through Nakagami imaging, the window-modulated compounding technique was used in this study. Details on the compounding Nakagami imaging algorithm based on the sliding window technique are reported by Tsui et al. [3, 13, 28].

**Fig 3:**
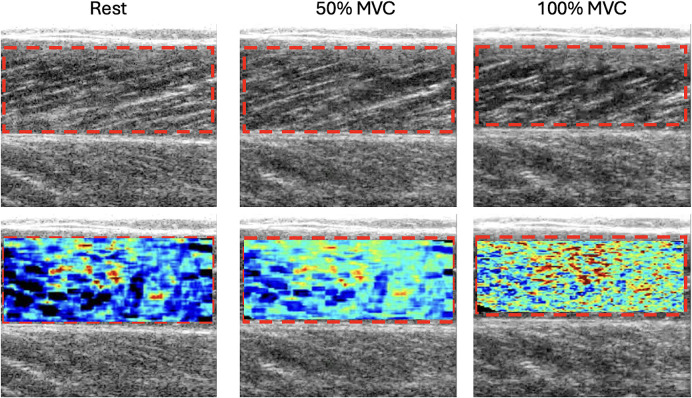
Upper row refer to the B-mode images and bottom row to the Nakagami images at rest, 50 and 100% of MVC. The region of interest (ROI) is highlightened in red.

All contractions were analyzed in the plateau of the force signal to obtain a representative value of Nakagami parameter at each contraction intensity. Therefore, at the end of these analyses we obtained 15 Nakagami parameter (three for each contraction intensity). An example of Nakagami images at rest, 50 and 100% of MVC are reported in Fig 3.

The intra-inter-operator reliability, as well as the between and within-days reliability, were not determined in this study but are expected to be high, as is generally the case for B-Mode evaluation.

### Statistical analysis

All data are reported as mean ± SD. Normality distribution of the data was assessed using the Saphiro-Wilk test. The main effect of intensity was estimated by means of a one-way repeated measure ANOVA. The interclass correlation coefficient (ICC; single method) was used to evaluate intra-trial reliability at each contraction intensity. The coefficient of variation (CV) was used as a measure of intra-subject variability. Repeated-measures Pearson correlations were performed using the “rmcorr package” to determine the within-subject association between Nakagami parameters (NA) and *k*. Statistical analysis was performed with Prism (GraphPad Prism 9.5.0) and Matlab (v.2021b). The alpha-level was set at 5%.

## Results

F_max_ was 774 ± 43 N and the actual values of force at the different loads corresponded to: 21±1.2%, 42±3.3%, 60±1.7%, 81±2.3%, and 100±2.4% of F_max_.

The results of the statistical analyses are reported in Table 1. For the Nakagami parameters, high values of intra-trial repeatability were observed at all the investigated intensities (range: 0.92–0.96) and the coefficient of variation was always lower than 10% (range: 8.7–9.6).

**Table 1:** Statistical results at each contraction intensity.

	*Contraction intensity (%Fmax)*				
	20%	40%	60%	80%	100%
**ICC**	0.92	0.94	0.96	0.93	0.94
**CV (%)**	9.3	9.6	8.7	8.9	9.3

Footnote

ICC and CV were calculated within each contraction intensity (three trials each)

**Fig 4:**
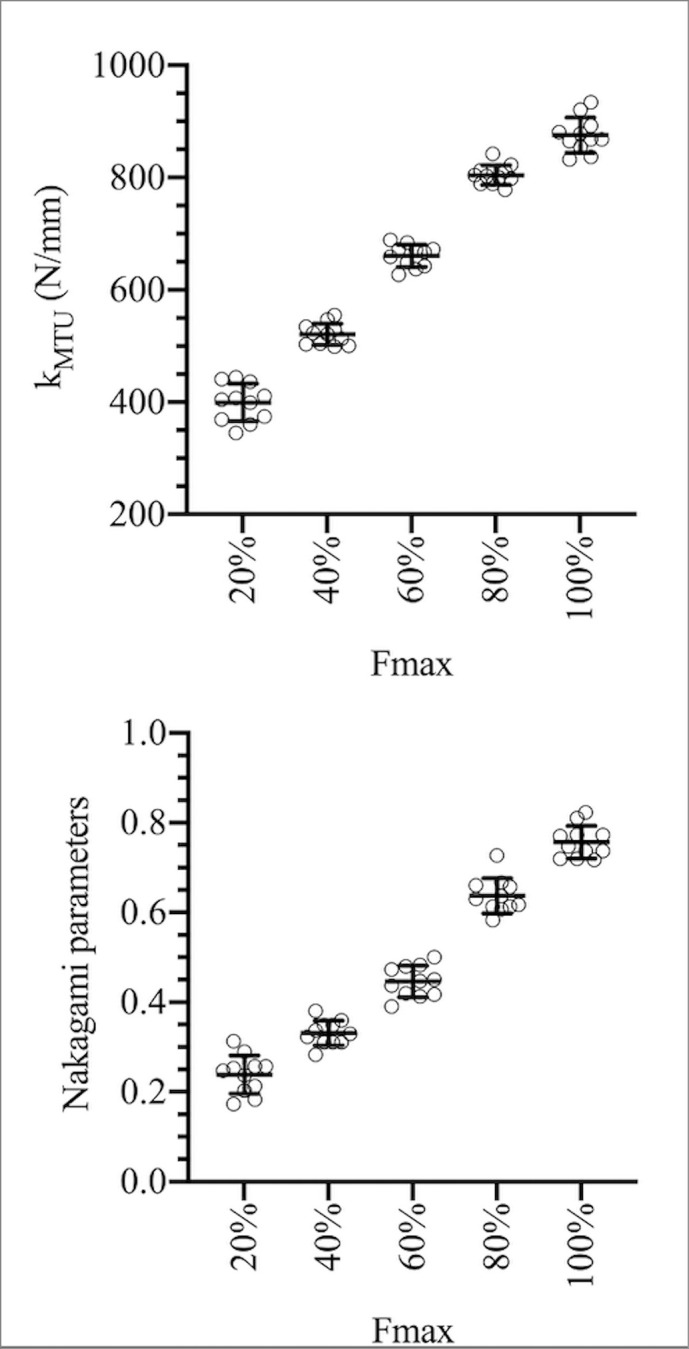
Individual values of stiffness (upper panel) and of the Nakagami parameters (the mean of the Nakagami values in the region of interest; lower panel) as a function of contraction intensity. Dots represent individual values; mean values (horizontal lines) and standard deviation (vertical bars) are also reported.

Individual values of stiffness and Nakagami parameters (the mean of the Nakagami values in the ROI) as a function of contraction intensity are reported in Fig 4. Both parameters were affected by contraction intensity, increasing as a function of force level (main effect: P<0.001 for both variables).

**Fig 5:**
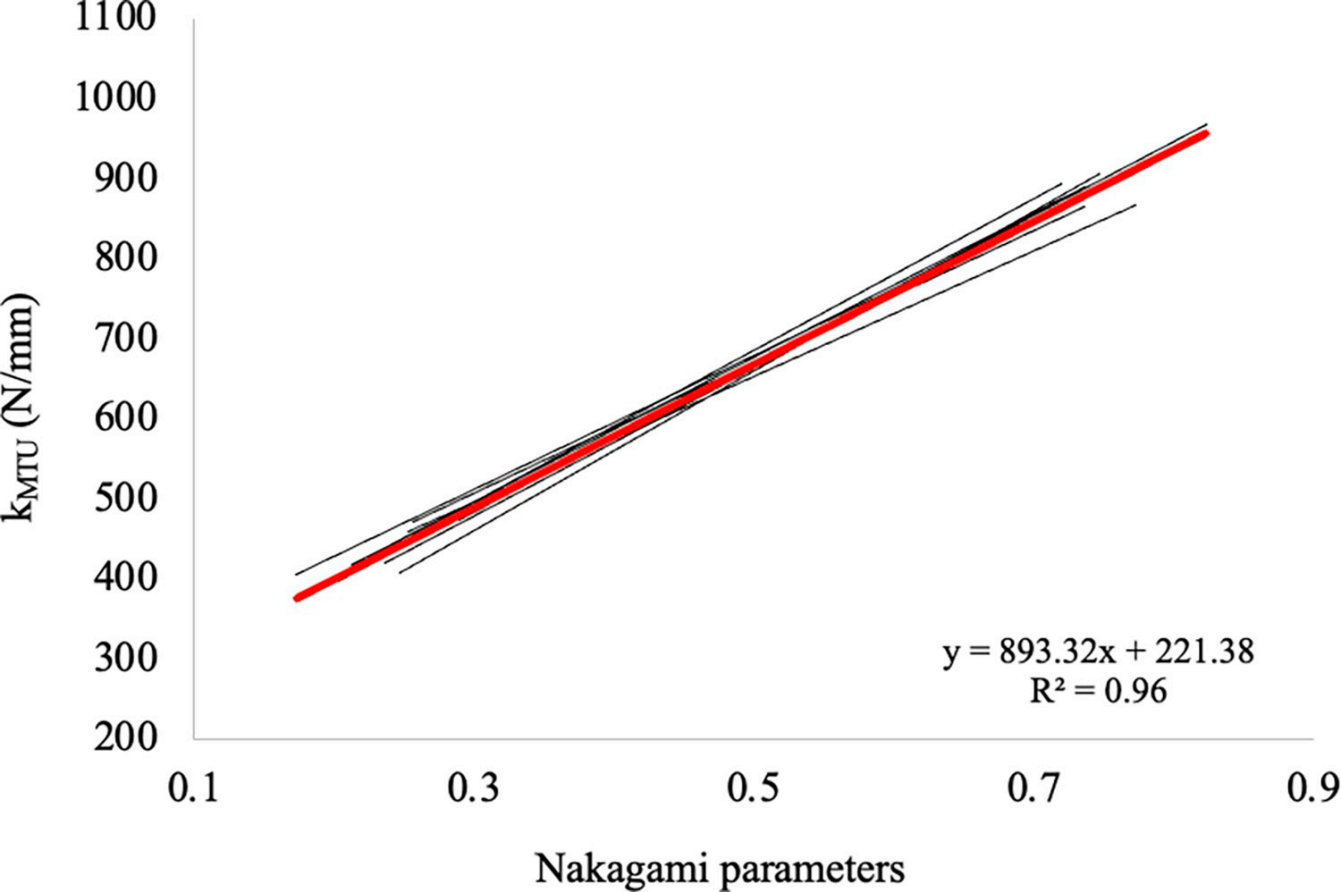
Relationship between stiffness values and Nakagami parameters for all subjects (thin black lines). Red line refers to the linear function (forced through zero) that represents all participants.

Significant repeated-measures linear relationships were observed between *k* and NA parameters in all subjects (P<0.001): the higher the values of muscle-tendon stiffness, the higher the NA parameters (Fig 5).

## Discussion

This study aimed to test the Nakagami transformation as a technique able to detect changes in mechanical properties (i.e., stiffness) of the investigated tissue. Specifically, we manipulated muscle-tendon unit stiffness during dynamic (and intense) fixed-end contractions while recording the behaviour of the NA parameters. We observed that Nakagami parameters increased linearly with the changes in muscle force (from 20 to 100% F_max_), as is the case for the values of stiffness assessed with the classic approach. Moreover, significant positive correlations were observed between the increases in *k* and in NA for all participants. These results indicate that the Nakagami technique does not saturate and thus provides a reliable tool to investigate the changes in a muscle’s mechanical properties during the whole contraction intensity range.

Muscle and tendon stiffness are important functional parameters related to sports performance, aging, disuse, and disease. For example, an increase in muscle stiffness (at rest, assessed by means of different methods) has been reported in people with Duchenne’s muscle dystrophy [13, 22], muscle fibrosis as well as in people affected by neurodegenerative disorders [4, 29]. In some of these studies, positive associations were observed between clinical scores and stiffness values, suggesting that evaluating muscle stiffness could have a clinical impact/significance, allowing the evaluation of the severity of this pathology.

However, the effects of a given pathology on locomotor function can be better evaluated during contraction, since an increase in stiffness impairs muscle’s dynamic shape changes. As an example [4], observed higher values of muscle-tendon unit (MTU) stiffness in people with Parkinson’s disease (with asymmetries between the more and the less affected side) and with a significant association with the clinical scores. To note, these data were collected during dynamic-high velocity contractions whereas in previous studies negligible differences in stiffness were observed in this population at rest (e.g. [30]).

When investigating an MTU in the contracted state, the choice of the US technique becomes important. Indeed, the classic approach requires a combination of ultrasound and dynamometric data and is not practical in a clinical environment. Data reported in this study indicate that the changes in Nakagami parameters were strongly related to the changes in *k*. In addition, this QUS technique is not affected by image saturation and can, thus, be applied at all contraction intensities, providing insight into the mechanical status of the investigated tissue.

While SWE is typically used to determine the intrinsic elastic characteristics of the tissue, ultrasound Nakagami imaging investigates the specific microstructure arrangement and can, thus, be utilized in combination with SWE to provide a more comprehensive evaluation of tissue properties [31]. For these reasons, recently released QUS software based on the Nakagami technique are now commercially available (e.g., Terason and SAMSUNG). Indeed, the construction of Nakagami images (based on Nakagami parameters) only requires RF data access from a standard B-mode system. Notably, the Nakagami images are derived from the backscattered signals produced by the tissue itself, making it largely independent of refraction signals from interfaces. For example, it was observed that Nakagami imaging is less affected by inflammatory activity compared to other quantitative ultrasound techniques, such as SWE.

Furthermore, the Nakagami images could be constructed by capturing RF data at a single time point. Given the frame rate of current clinical systems, motion impacts on Nakagami parameters could be minimal, as timely data acquisition is ensured. The Nakagami parameters are also relatively unaffected by possible confounding variables, such as lesion location and type of tissue [32].

Another point in favour of the Nakagami technique is that this modality offers information on tissue microstructures [16]. Fat infiltration and fibrosis formation were observed to increase the amplitude of the backscattered signal (and therefore the Nakagami parameters) in liver fibrosis; this suggests that ultrasound backscattering may provide useful information regarding changes in muscle microstructures too. This assumption fits with our data, because an increase in MTU stiffness as a function of contraction intensity could be related to an increase in intramuscular pressure and to a modification of the MTU structures [33–35].

This should be the topic of future studies since, to our knowledge, this is the first study where the Nagakami technique was applied (and validated) to investigate the muscle in the contracted state.

### Limitations and further considerations

Nakagami parameters share common limitations with other QUS techniques. First, the sensitivity of NA parameters depends on the size of the transducer resolution cell (i.e., the focusing effect) [36]: the higher the ultrasound frequency to measure backscattered signals, the higher the sensitivity in detecting changes in tissue properties. Second, the statistical nature of the acquired signals for estimating the Nakagami parameter needs to follow the Nakagami distribution [37]. Although previous studies demonstrated that ultrasound backscattered signals are Nakagami-distributed, future work should consider more flexible statistical analysis approaches for envelope statistics imaging of muscle regardless of the statistics of backscattered signals, such as information entropy imaging [38, 39]. Third, ultrasound scanning orientation could be a confounding factor for muscle evaluation. Since the muscle is an anisotropic tissue, the imaging plane (e.g., transversal or longitudinal) of ultrasound Nakagami imaging could affect data interpretation. Moreover, as long as the transducer is placed on the skin without inducing significant deformation/changes in the tissue’s elastic properties, reliable Nakagami imaging data can be obtained also for muscle tissues [32].

The present study compared the behavior of the Nakagami parameters as a function of contraction intensity with the behavior of the “muscle-tendon stiffness”. This term is generally used because the measured displacement of the muscle belly reflects, besides the elongation of the muscle itself, also the combined elongation of the distal aponeurosis and free tendon [5, 40]. In order to determine muscle stiffness per se, other experiments should have been performed (e.g. quick release method, by using instrumentations such as the Biodex).

In this regard, we determined the ROI of the RF analysis by excluding the aponeuroses, as proposed in the few other studies where the muscle was investigated [22]. Since Nakagami parameters are sensible to the microstructural characteristics of the tissue, future studies should check the effect of including the aponeuroses in the ROI on NA parameters.

In this study, only VL behavior was investigated but, of course, the quadriceps force is not generated by VL alone. We could have calculated the relative contribution of the VL PCSA within the PCSA of the quadriceps by using a scaling factor of the whole muscle torque/force, but this procedure is not expected to influence the behavior of muscle force as a function of contraction intensity.

## Conclusion

Nakagami parameters increase as a function of contraction intensity following the behaviour of muscle-tendon stiffness during contraction. Our data suggest that the change in Nakagami parameters reflect changes in tissue stiffness due to a modification of their microstructures. Last but not least, we observed that Nakagami parameters could be a valid and reliable tool to investigate the changes in muscle-tendon stiffness during contraction across the entire range of contraction intensity, making this technique a valid tool to investigate changes in musculoskeletal mechanics.

## Supporting information

S1 Data (XLSX)
